# Hypertransfusion Therapy in Sickle Cell Disease in Nigeria

**DOI:** 10.1155/2014/923593

**Published:** 2014-08-07

**Authors:** Ademola Samson Adewoyin, Jude Chike Obieche

**Affiliations:** Department of Haematology and Blood Transfusion, University of Benin Teaching Hospital, PMB 1111, Benin City, Edo State, Nigeria

## Abstract

*Introduction*. Hypertransfusion refers to chronic blood transfusion therapy aimed at ameliorating disease complications in various haemopathies particularly the haemoglobinopathies. In sickle cell disease, hypertransfusion is aimed at maintaining patient's haemoglobin level at 10 to 11 g/dL using haemoglobin AA blood and its resultant dilutional effect on sickle haemoglobin is sustained by intermittent long-term transfusions. *Aim and Objective*. This paper highlights hypertransfusion and its privileged position as a secondary measure in prevention and treatment of sickle cell disease, especially in the Nigerian context. *Materials and Methods*. Relevant literatures were searched on PubMed, Google Scholar and standard texts in haematology and transfusion medicine. Keywords used in the search are hypertransfusion, sickle cell disease, chronic transfusion, and Nigeria. Literatures gathered were reviewed, summarized, and presented in this paper. *Result*. Immense clinical benefit is associated with hypertransfusion therapy including prevention of stroke and amelioration of severe sickle cell disease especially in transplant ineligible patients. Careful patient selections, appropriate blood component, and prevention of transfusion hazards as well as oversight function of an experienced haematologist are pertinent to a successful hypertransfusion therapy. *Conclusion*. Improved knowledge of the benefits and practice of hypertransfusion will effectively translate into improved health status even among Nigerian sickle cell disease patients.

## 1. Introduction

Sickle cell disease (SCD) is the commonest monogenetic disease worldwide and its greatest burden is found in Sub-Saharan Africa especially Nigeria [1, 2]. In most parts of West Africa, the prevalence of sickle cell trait ranges between 10 and 40% of the population [3]. However, in some areas in Uganda, prevalence has been reported to be as high as 45% while in Nigeria about 20 to 30% of the population are trait carriers [4, 5]. A recent survey in South-south Nigeria revealed that about 2.39% of the population is affected by sickle cell disease [6]. In Africa, significant morbidity and mortality are still associated with the disease, hence the need for effective and definite control measures [7, 8].

Care and treatment of SCD patients require expertise commitment, as well as a wide array of therapeutic and prophylactic measures including adequate analgesia, anticoagulation as indicated, transfusion of requisite blood components when necessary, haemopoietic stem cell transplantation, oxygen therapy when needed, routine prophylactic medications (antimalarial, multivitamins supplements, low dose aspirin, and antioxidants), hydroxyurea therapy, adequate hydration, and immunization against infectious pathogens especially in early childhood. Moreover, care of SCD patients requires a multispecialist team including haematologists, orthopedic surgeons, plastic surgeons, urologists, nephrologists, specialist nurses, counselors, and medical social workers [9].

Carrier detection and genetic counseling have been recommended by World Health Organization for the control of SCD [10]. In Nigeria, efforts at public education on sickle cell disease and its prevention as well as carrier detection with genetic counseling have not made sufficient far reaching improvements in sickle cell disease control. There is still a palpable dearth of public knowledge about sickle cell disease [11–14]. Studies have also shown the option of selective abortion following prenatal diagnosis to be unacceptable to a significant proportion of persons [15, 16]. At the primary prevention level, there are currently no functional nationwide neonatal screening policies or programs for early detection and optimal treatment. Though it is expected that a country like Nigeria with very high prevalence of sickle cell trait should have a nationwide program at all levels of care for control and care of sickle cell disease, available evidence suggests that effective control of SCD in Nigeria is still largely infantile.

Apart from premarital counseling and continuous medical education, a number of other strategies including chronic transfusion have been scientifically proven to reduce complications and improve quality of life in SCD patients. This work therefore is aimed at collecting and summarizing available information on the practice and utilization of hypertransfusion in the care of SCD patients in Nigeria.

## 2. Hypertransfusion Therapy in Sickle Cell Disease

Hypertransfusion refers to a chronic blood transfusion regimen with therapeutic intention of reducing sickle haemoglobin levels over a long period of time [17, 18]. Generally, blood transfusion in medicine entails safe transfer and infusion of blood components into an individual to meet specific physiological needs. Such needs include improving haemoglobin level for better tissue oxygenation, replacement of deficient coagulation factors, and platelet transfusions [19, 20]. Transfusion modalities in SCD include top up (simple), exchange, and chronic blood transfusions. The different transfusion plans have specific benefits in various clinical scenarios in SCD.

Traditionally, transfusion in sickle cell disease was carried out only in emergencies until the early 1980s when the benefits of chronic transfusion began to gain clinical appreciation [21]. Approach to hypertransfusion may be the conservative transfusion therapy aimed at maintaining haemoglobin level around 10 to 11 g/dL with about 50% reduction in haemoglobin S level or the aggressive approach aimed at reducing sickle haemoglobin level below 30% [22]. The goal of hypertransfusion in sickle cell disease is to reduce the level of circulating haemoglobin S below a target level for prevention and treatment of disease complications associated with sickle haemoglobinopathies. Red cell transfusion also serves to correct the attendant anaemia in SCD, suppress endogenous erythropoiesis, and reduce haemolysis. Hypertransfusion has been found to effectively mitigate the complications of sickle cell anaemia in some randomized studies [23–28]. It is well-established that there is a high correlation between disease manifestations and level of circulating haemoglobin S [29]. The bulk of the disease burden is due to high percentage of sickle haemoglobin within the red cells and the plasma sickle haemoglobin levels.

As a rule, transfusion therapy in sickle cell disease is carried out with haemoglobin AA blood. Besides reducing the level of circulating haemoglobin S, exogenous supply of haemoglobin AA red cells also suppresses endogenous erythropoiesis, thereby suppressing intrinsic sickle haemoglobin levels. The primary event in sickle cell disease pathogenesis is deoxyhaemoglobin precipitation. Normal red cells contain about 200–300 million soluble haemoglobin A molecules. However, red cells in sickle cell disease contain high level (80–90%) of the less soluble sickle haemoglobin which crystallizes out of solution under low oxygen tension. Under conditions of low oxygen tension (PaO_2_ less than 35–40 mmHg), haemoglobin S molecules undergo nucleation with progressive polymerization of haemoglobin molecules and eventual crystallization into tactoids with seven double strands which cross-links. This gives rise to the crescent shape appearance of the sickled red cells on blood cytology. With reoxygenation, the polymers dissolve. However, with repeated deoxygenation and reoxygenation in different vascular beds, the red cells permanently acquire a sickle shape. This is termed irreversible sickle red cells (ISCs) when seen on peripheral blood smear. Again, the percentage of ISCs has been shown to correlate directly with severity of haemolysis and inversely with frequency and severity of vasoocclusive crisis [30–32].

At the molecular level, there is substitution of valine for glutamate at position 6 of the beta sickle haemoglobin chain due to the single base change from adenine to thymidine. The resultant HbS exposes a hydrophobic patch under low oxygen tension and interactions between these patches lead to nucleation and eventual polymerization [30]. ISCs are less deformable and more prone to fragmentation which results in intravascular haemolysis. High circulating level of sickle haemoglobin causes significant pathology in sickle cell disease. When released, tetrameric haemoglobin dissociates into dimers with molecular weight of 34 kDA. Free haemoglobin, being an avid scavenger of nitric oxide (NO), leads to reduction in NO bioavailability. Lysis of red cells releases the arginase-1 which breaks down arginine, a metabolic substrate required in biosynthesis of NO. This further contributes to reduction in NO synthesis and further depletion of plasma NO levels. Vasospasm in sickle cell disease results from marked depletion of NO, a potent vasodilator. Defect in ornithine pathway favors alternate pathways with increased production of prolines and polyamines. These byproducts induce smooth muscle proliferation; the resultant proliferative vasculopathy produces disease complications such as pulmonary hypertension, retinopathy, cerebrovascular disease, and chronic leg ulcers. Some other disease manifestations such as bone pain crisis and osteonecrosis are associated with heightened blood viscosity and microvascular occlusion by the irreversibly sickled red cells. Deformable sickle cells are known to express CD36 and very late antigen 4 (VLA4). CD36 and VLA4 interact with vascular endothelium via thrombospondin and VCAM-1, respectively, leading to vasoocclusion with subsequent ischaemic injury and organ infarctions [22, 29]. In line with the current understanding of the pathophysiology of SCD, disease manifestations and complications can therefore be averted by treatment protocols aimed at reducing the circulating ISCs, plasma sickle haemoglobin levels, and therapeutic suppression of the disordered host erythropoiesis in these patients. Thus, hypertransfusion is currently indicated in treatment of SCD as further discussed below.

### 2.1. Clear Indications for Use of Hypertransfusion Therapy [33–41]

- Prevention of first stroke (cerebrovascular disease).
- Prevention of repeat stroke.
- Transcranial Doppler (TCD) ultrasonography with cerebral blood flow > 2 m/sec (highly predictive of stroke).
- Delayed growth and development in children.
- Frequent acute chest syndrome unresponsive to hydroxyurea.
- Frequent severe bone pain crisis requiring three or more hospital admissions per annum and unresponsive to hydroxyurea therapy.
- Severe SCD lacking HLA-matching donor.
- Sickle chronic lung disease.
- Chronic vital organ failure.
- Pregnant women with bad obstetric history and frequent bone pains.

### 2.2. Relative Indications [25, 40, 42]

- Sickle cell leg ulcers (to improve tissue oxygenation and wound healing and reduce vasculopathy).

### 2.3. Controversial Indications [35, 40]

- Recurrent sickle cell priapism.
- Preparation for infusion of contrast media.
- “Silent” cerebral infarct and/or neurocognitive damage.

### 2.4. Inappropriate Indications [35]

- Steady state (compensated anaemia).
- Uncomplicated pain episodes.
- Infections.
- Minor surgery that does not require general anaesthesia.
- Aseptic necrosis of the hip or shoulder (unless indicated for surgery).
- Uncomplicated pregnancy.

### 2.5. Relative Contraindications [36]

- Multiple red cell alloantibodies.
- Poor venous access.

### 2.6. Suggested Guidelines on Hypertransfusion

- Decision to initiate a chronic hypertransfusion regimen in a patient should be individualized. Benefit to risk should be carefully assessed and a clear indication must be present.
- Decision to hypertransfuse should be communicated to the patient and relatives in clear terms (stating the benefits and potential adverse effects) and informed consent should be obtained.
- Design a clearly written therapeutic plan and all clinical staff must be duly communicated.
- Blood bank must be duly informed and necessary pretransfusion services commenced.
- Prehypertransfusion laboratory work-up should include: blood type with extended red cell phenotyping, antibody screening for unexpected antibodies if indicated, serum ferritin levels, screening for hepatitis A, B, and C, retroviral disease status, liver function test, electrocardiogram/echocardiography, and possibly audiologic/ophthalmologic examinations [35, 43].
- Therapeutic goals such as the final (posttransfusion) haematocrit and the target sickle haemoglobin level should be set before each transfusion episode.
- On the average, most patients will require 2-3 units every 4–6 weeks. As such, transfusion requirement should be established in each individual patient and monitored as changes may occur with time [44].
- Rapid transfusion should be avoided to prevent hyperviscosity. Overtransfusion or supertransfusion (where haemoglobin level is raised above 11 g/dL) should be avoided [29].
- Transfusion requirement will vary from patient to patient depending on quality of the donor unit, associated morbidities, and biologic variables in the patients.
- The aim is to keep the haematocrit below 35% to prevent hyperviscosity [29, 33, 45]. Increased viscosity may precipitate hypertransfusion syndrome if haematocrit is elevated too rapidly or haematocrit is greater than 35% [29].
- If the pretransfusion haematocrit is >35%, chances are that hyperviscosity may be contributing to acute complications like acute chest syndrome, stroke, and chronic complications like chronic pain syndrome, osteonecrosis, and ocular disease [26]. Such patients should have one or two units removed, followed by repeated exchange transfusions until desired sickle haemoglobin reduction is achieved [29].
- The patient should report to the outpatient clinic or day-care unit at least 24 hours before each planned transfusion for a full blood count, reticulocyte count, and initial sickle haemoglobin level.
- Formula for estimation of required transfusion volume and its dilutional effect of transfusion on HbS levels are as follows [29, 34]:
  - simple red cell transfusions: PRBCV (mL) = [(HCT_*d*_   −  HCT_*i*_)/HCT_rp_] × TBV,
  - dilutional effects of transfusion on Hb S levels: Hb  S_f_(%) = {1 − [(PRBCV × HCT_rp_)/(TBV × HCT_*i*_) + (PRBCV × HCT_rp_)]}   ×  Hb  S_*i*_,
  - manual partial exchange transfusion: exchange volume (mL) = [(HCT_*d*_   −  HCT_*i*_) × TBV]/[HCT_rp_   −  {(HCT_*i*_ + HCT_*d*_)/2}],
  - automated exchange transfusion: red cell volume (mL) = HCT_*i*_   ×  TBV, where
  - PRBCV: packed red blood cell volume,
  - HCT_*d*_: desired haematocrit,
  - HCT_*i*_: initial haematocrit,
  - TBV: estimated total blood volume in mL (children 80 mL/kg, adults 65–70 mL/kg),
  - Hb  S_*f*_: final Hb S levels,
  - HCT_rp_: haematocrit of replacement cells (usually 0.7 to 0.8),
  - Hb  S_*i*_: initial Hb S levels.
- Patient should be immunized for hepatitis B and C. Booster doses should be given annually to maintain good antibody levels [41].
- Monitor for iron overload and commence s/c desferrioxamine infusion when serum ferritin > 1000 ug/L or >20 units of red cell concentrate has been administered [36]. Oral iron chelators like deferasirox (exjade, asunra) are locally available.
- Overall, the managing physician should ensure that the right blood and the right amount are administered to the right patient at the right time in the right place.

### 2.7. Potential Hazards of Hypertransfusion

The art of blood transfusion is not without potential hazards to its recipients. Awareness of complications associated with blood transfusion and hypertransfusion (in particular) helps to position the clinician with strategies to keep these untoward effects to the barest minimum. Hazards of blood transfusion are vast and may be categorized as acute or chronic, immunologic or nonimmunologic. They may also be categorized as early (arising within 24 hours of commencement), delayed (up to 4 weeks), or long-term. Early complications include allergic reactions, anaphylaxis, febrile nonhaemolytic transfusion reaction (FNHTR), acute haemolysis, volume overload, hypothermia, metabolic derangements including hyperkalaemia, hypocalcaemia, and acid-base disturbances, transfusion related acute lung injury, thrombophlebitis, citrate toxicity, bacterial contamination, air embolism, and clotting abnormalities. Late complications include delayed haemolysis, alloimmunization, transfusion associated graft versus host disease, iron overload, transfusion transmissible infections, post transfusion purpura, and transfusion associated immune-modulation. Suffice to say, red cell alloimmunization and iron overload are peculiar complications of chronic blood transfusion in SCD.

Generally, the most frequent transfusion hazard is febrile nonhaemolytic transfusion reaction [46, 47]. Usually, this is caused by exposure of an alloimmunized recipient to foreign antigens on donor leucocytes and platelets leading to the release of pyrogens such as IL-1 and TNF-alpha. Also, leakage of cytokines from inflammatory cells in the stored blood has been proposed to cause FNHTR. Risk of FNHTR is higher with multiply transfused patients and in multiparous women. FNHTR usually begins within thirty minutes to one hour of transfusion and manifest with fever, chills, headache, or itching. Treatment is to discontinue transfusion, exclude other causes of fever such as bacterial contamination and haemolytic reaction, underlying disease in the patient, and administer antipyretics and antihistamine. Leucodepleted red cell, premedication with antipyretic, and slow speed of transfusion are preferable in subsequent transfusions.

Acute haemolysis (AHTR) is the most dangerous transfusion reaction. It is usually due to incompatible blood components from clerical errors. Transfusion of incompatible units leads to immune response and activation of complement cascade leading to intravascular haemolysis. Also massive release of inflammatory cytokines (cytokine storm) and anaphylatoxins leads to hypotension and acute renal failure. Severe intravascular haemolysis can trigger disseminated intravascular coagulopathy and fatality may ensue. Acute haemolytic transfusion reaction (AHTR) is an emergency. Usually, AHTR begins within few minutes of starting the transfusion. Conscious patients complain of pain or heat at the infusion site, restlessness (akathisia), and loin pain. Fever develops with associated chills and rigor, tachycardia, hypotension/shock, and bleeding tendencies. Hypotension and oozing from venipuncture sites may be the only signs in an unconscious patient.

In event of a suspected AHTR, transfusion should be stopped immediately. Then, maintain plasma volume with crystalloids and manage complications that may arise. Haemovigilance unit should be notified immediately. Investigation of AHTR includes checks for haemolysis (visual examination of patient's plasma and urine, spherocytosis on blood film, increased serum bilirubin, and LDH levels), checking the compatibility form, blood label and patient's identity, repeat blood grouping of recipient pre- and posttransfusion blood sample and on donor's blood unit, repeat cross-matching of donor blood against recipient's pre- and posttransfusion samples, direct antiglobulin test on pre- and posttransfusion samples, run coagulation profile, D-dimer to rule out DIC, and finally electrolyte/urea/creatinine to rule out acute renal failure [48].

Urticarias are due to allergens (usually plasma proteins) in the donor blood to which the recipient has been previously sensitized. Patient develops rashes and pruritus within minutes of transfusion. Treatment is to slow the transfusion rate and administer antihistamine. if patient is unresponsive to antihistamines, discontinue transfusion. Anaphylaxis is a form of severe allergy, quite rare, and is associated with immunoglobulin-A deficient recipients. Infusion of immunoglobulin-A containing blood component into the recipient triggers the formation of IgA/anti-IgA aggregates with the activation of alternate complement pathway. Release of anaphylatoxins (C5a and C3a) mediates anaphylaxis. Transfusion should be stopped immediately and patient is given adrenaline, chlorpheniramine/promethazine, and hydrocortisone. Hypothermia, metabolic derangements (hyperkalaemia, hypocalcaemia, and acid-base imbalance), citrate toxicity, and clotting abnormalities are associated with large volume transfusions and are unlikely in hypertransfusion therapy. Thrombophlebitis may occur as in any condition warranting insertion of a peripheral or central venous catheter. Peripheral line should be changed every 3-4 days and removed when not in use.

Delayed haemolysis is an immunological reaction that occurs in alloimmunized individuals with low (undetectable) antibody titre which is often missed during compatibility testing. Implicated antibodies include non-D Rh (E, C, and c), kell, duffy, and kidd antibodies [49, 50]. On reexposure to the antigen, a secondary (anamnestic) immune response ensues with massive antibody production, manifesting about 5 to 10 days later with fever, jaundice, and declining haemoglobin levels (incongruent with expected haemoglobin rise). Usually, it is less severe than AHTR and the haemolysis is extravascular. Antibody screening and identification are important. Least incompatible blood is indicated for subsequent transfusions.

Transfusion associated graft versus host disease (GvHD) is associated with immune-compromised recipients. It results from immune attack of recipient tissues by immune-competent donor T lymphocytes. Blood components for immune-compromised persons should be irradiated (25 Gy) before use.

With proper donor selection blood screening for pathogens, the risk of transfusion transmissible infection (TTI) is negligible. However, in developing nations such as Nigeria, TTIs still pose a major challenge. International standards such as predonation questionnaire and ELISA based TTI screening are yet to become a routine in many blood banking facilities. In addition, there is poor haemovigilance reporting; as such there is little or no data for monitoring and evaluation of transfusion services.

Alloimmunization to donor red cell antigens and iron overload from repeated transfusions poses a serious challenge to effective hypertransfusion therapy. One unit of red cell concentrate contains about 200–250 mg of iron. Daily physiological loss of iron (through desquamation of skin and mucous membrane) is only about 1 mg. As such, the repeated transfusions and heightened haemolysis in sickle cell disease create a positive iron balance, leading to transfusion siderosis over time. Excess iron in the body will get deposited in virtually every organ in the body, most especially the heart, liver, skin, and endocrine organs and gonads. That would lead to heart failure, diabetes mellitus, skin pigmentation, and gonadal failure. It is advised that iron status of such patients should be monitored every 6 months. Iron chelation therapy should be commenced early when liver iron store exceeds 7 mg/g in adults or 4 mg/g in children [36, 51]. Another useful indicator is to begin iron chelation after cumulative transfusion of 120 mL/kg of red cell concentrate which equates to transfusions in excess of 20 to 30 units of red cells [36]. Serum ferritin levels greater than 1000 ng/mL can be used in steady state but can be quite unreliable. Serum ferritin is an acute phase reactant and is falsely elevated in inflammations, liver disease, and high vitamin C stores. Compared with standard transfusion therapy, erythrocytapheresis greatly reduces the risk of iron overload but the cost is more.

Recipient alloimmunization is another serious problem that occurs with multiple transfusions. It reduces the chances of a successful transfusion at subsequent times. As a rule, patients being planned for hypertransfusion should have an extended red cell typing to identify other clinically significant blood group antigens including Rh, kell, kidd, and duffy [42, 43, 52]. As much as possible, a group of identical blood units should be transfused. For patients already alloimmunized, antigen negative or the least incompatible blood unit should be transfused. Studies show that 18 to 36% of multiply transfused sickle cell anaemia patients become alloimmunized [53, 54]. Some studies have reported a lower incidence of alloimmunisation with a closer donor-recipient matching for minor red cell antigens and race [54, 55]. However, a recent study of 182 patients with SCD who received antigen matched (phenotype matching for Rh-D, C, E, and K antigen) and racially matched blood revealed a significant alloimmunization rate of 58% and 15% for those received chronic transfusions and episodic transfusions, respectively [56]. Findings from this study suggest that little benefit may be associated with racial and phenotypic red cell matching possibly due to Rh variants that are not detected by red cell phenotyping [56]. Genotypic matching of red cell concentrate may, however, be more beneficial. Further research is required to clarify these positions. Chronic blood transfusion has been associated with hypersplenism and splenomegaly, which may result in increased transfusion requirements over time [57].

## 3. Current Practice, Challenges, and Prospects of Hypertransfusion in Nigeria

Predating this paper, I found sparse local data on the prevalence and pattern of blood use in Nigerian SCD population. Barbara Otaigbe recently reported a high rate of blood use among paediatric SCD patients in south-south Nigeria and noted the commonest indication for simple transfusions to be severe anaemia [58]. However, no report was made on the use of hypertransfusion in the study [58]. However, in a recent report by Oniyangi et al., chronic transfusion therapy was used in prevention of recurrent stroke among some SCD children in Abuja and it showed some beneficial effects [59].

Furthermore, there is dearth of scientific data on the level of awareness, knowledge, and practice of hypertransfusion in SCD among Nigerian general duty doctors and specialists alike. As such, its current challenges may include poor awareness, poor knowledge, and lack of technical expertise among health care givers. Insufficient supply of blood and blood components due to inefficient blood banking services even at tertiary health care levels is a major challenge in our blood banks [60, 61]. Also, the financial burden of the therapy to the patient, the family, and the society at large is quite heavy as most of the blood supply is commercially driven or at best family replacement donations [60, 61]. Procurement of blood from commercial vendors further increases the risk of transfusion acquired infections and cost of transfusion services which has to be borne by the patient and caregivers. Recently, Ejeliogu et al. reported that HIV transmission is still transmissible through blood transfusion in Nigerian patients with SCD especially commercially sourced units transfused in peripheral hospitals [62]. In another recent report, Lagunju et al. reported a low acceptance rate of chronic transfusion therapy among caregivers of SCD children in Ibadan [63]. Major reasons for decline included its high cost, unavailability of blood, and the need to regularly seek for donors. The mean cost of chronic blood transfusion (excluding chelation therapy) was found to be 3,276 US Dollars (SD = 1,168) per annum [63].

Moreover, the cost of instituting iron chelation therapy adds to the overall cost of hypertransfusion therapy. Invariably, hypertransfusion therapy may cost beyond the reach of most eligible Nigerian SCD patients. The option of erythrocytapheresis is relatively inaccessible and unaffordable. Thus, the practice of hypertransfusion in Nigeria may be bewildered by suboptimal transfusion services and complications such as monitoring and treatment of iron overload. It behooves us to say that its successful use in the Nigerian context cannot be disconnected from general improvements in our national transfusion service. As well, specialized supports including financial aids, trained professionals, and comprehensive sickle cell centers/clinics should be dedicated to treatment of SCD. Better government commitment to the treatment and prevention of sickle cell disease is necessary to expedite proper healthcare delivery to affected persons.

## 4. Conclusion

Hypertransfusion is an effective disease modifying strategy in the management of SCD patients. Despite its applicability, there is still little data on its utilization in Nigeria. The reason for the scarcity of information on this treatment modality could be nonusage of the strategy, low acceptance rate, and/or poor attitude to documentation and analysis of such data.

Considering the potential benefits of chronic blood transfusion on quality of life and overall survival in sickle cell disease, we advocate that selected Nigerian patients with adequate resources should be offered hypertransfusion following proper patient education and informed decision on its potential benefits, complications, and cost of therapy. Apheresis for red cell exchange should be provided, accessed, and used when indicated.

Other disease modifying strategies in management of sickle cell disease such as haemopoietic stem cell transplantation are either not readily available or not affordable in developing countries. Therefore, this paper serves as a wake-up call to physicians in Nigeria to practice and promote judicious use of hypertransfusion therapy.

The term hypertransfusion is not exclusive to treatment of SCD alone. There are other clinical indications that may warrant a chronic transfusion regimen, conditions such as major thalassaemia, refractory myelodysplastic syndrome, and aplastic anaemia. However, the strategic therapeutic choice for hypertransfusion in SCD is freshly donated (less than 24 hours), sickle negative, leucodepleted, phenotypically matched, cytomegalovirus negative, and perhaps a minority and racially matched red cell concentrate.

Further efforts should be directed at educating professionals involved in sickle cell disease management at all levels of healthcare to boost their technical knowledge and expertise. Amongst the few disease-modifying interventions that are currently available in SCD care, only haemopoietic stem cell transplantation (HSCT) is potentially curative. However, because of its potential toxicities, HSCT tends to be employed more readily in patients with severe sickle cell disease aged less than 16 years and having a matched sibling donor [64]. Gene therapy offers great hope of cure theoretically but effective vector for stem cell gene transfer is yet to be designed [65]. Hydroxyurea therapy has also been shown to significantly improve outcome in SCD [66–68]. Still yet, hypertransfusion remains a cornerstone strategy especially in those that are unresponsive to hydroxyurea or when hydroxyurea is contraindicated. With effective practice of hypertransfusion, patients with severe sickle cell disease can live a near normal life, with significant reduction of disease morbidity and mortality.
